# The *Defective in Autoregulation (DAR)* gene of *Medicago truncatula* encodes a protein involved in regulating nodulation and arbuscular mycorrhiza

**DOI:** 10.1186/s12870-024-05479-6

**Published:** 2024-08-10

**Authors:** Elise Schnabel, Sagar Bashyal, Cameron Corbett, Tessema Kassaw, Stephen Nowak, Ramsés Alejandro Rosales-García, Rooksana E. Noorai, Lena Maria Müller, Julia Frugoli

**Affiliations:** 1 Department of Genetics and Biochemistry, Clemson University, Clemson, SC 29634 USA; 2 Department of Biology, University of Miami, Coral Gables, FL 33124 USA; 3 Department of Biological Sciences, Clemson University, Clemson, SC 29634 USA; 4 Clemson University Genomics and Bioinformatics Facility, Clemson University, Clemson, SC 29634 USA; 5 Plant Molecular and Cellular Biology Laboratory, Salk Institute for Biological Studies, La Jolla, CA 92037 USA; 6 School of Biological Sciences, University of California San Diego, San Diego, CA 92093 USA; 7 Present addresses: Department of Biology, West Virginia University, Morgantown, WV 26506 USA; 8 Present addresses: Department of Biology, Colorado State University, Fort Collins, CO 80523 USA; 9 Present addresses: Center for Technology Licensing, Cornell University, Ithaca, NY 14850 USA

**Keywords:** Autoregulation of nodulation, AON, Autoregulation of mycorrhizal symbiosis, AOM, DAR, Hypernodulation, *Medicago truncatula*

## Abstract

**Background:**

Legumes utilize a long-distance signaling feedback pathway, termed Autoregulation of Nodulation (AON), to regulate the establishment and maintenance of their symbiosis with rhizobia. Several proteins key to this pathway have been discovered, but the AON pathway is not completely understood.

**Results:**

We report a new hypernodulating mutant, *defective in autoregulation*, with disruption of a gene, *DAR* (*Medtr2g450550/MtrunA17_Chr2g0304631*), previously unknown to play a role in AON. The *dar-1* mutant produces ten-fold more nodules than wild type, similar to AON mutants with disrupted *SUNN* gene function. As in *sunn* mutants, suppression of nodulation by CLE peptides MtCLE12 and MtCLE13 is abolished in *dar*. Furthermore, *dar-1* also shows increased root length colonization by an arbuscular mycorrhizal fungus, suggesting a role for DAR in autoregulation of mycorrhizal symbiosis (AOM). However, unlike *SUNN* which functions in the shoot to control nodulation, *DAR* functions in the root.

**Conclusions:**

*DAR* encodes a membrane protein that is a member of a small protein family in *M. truncatula*. Our results suggest that DAR could be involved in the subcellular transport of signals involved in symbiosis regulation, but it is not upregulated during symbiosis. *DAR* gene family members are also present in Arabidopsis, lycophytes, mosses, and microalgae, suggesting the AON and AOM may use pathway components common to other plants, even those that do not undergo either symbiosis.

**Supplementary Information:**

The online version contains supplementary material available at 10.1186/s12870-024-05479-6.

## Background

Legume plants benefit from the ability to fix nitrogen (N) from the air when grown in low N conditions. In the presence of compatible species of rhizobia, legumes establish a symbiotic relationship with rhizobia, housing the bacterial cells in de novo organs called nodules. Rhizobia have the ability to fix N from the atmosphere using the nitrogenase enzyme exclusive to prokaryotes and make it available to the plant in exchange for carbon compounds. The process of nodulation is tightly regulated by the plant, as hosting the symbiont and the nitrogenase reaction requires maintaining carbon and N homeostasis as well as controlling oxygen tension in the nodules. *Medicago truncatula* and other legumes regulate the formation of nodules and the number that form through at least three pathways depending on N demand, availability and rhizobial compatibility [1]. One pathway, the N demand pathway, regulates nodulation in response to available nitrogen, allowing nodulation when N is scarce and limiting nodulation when N is abundant. A second pathway regulates nodulation based on compatibility at the initiation of contact between the organisms, and a third pathway, the Autoregulation of Nodulation (AON) pathway, regulates the development of the nodules that will host the rhizobia based on the number of already existing nodules [1]. The N demand sensing pathways appear to be conserved not just in legumes but also in most other plants [2]. The AON pathway receives input from the N demand pathway as well as other signals to control the number of nodules formed on the plant [3–6]. AON is both local and systemic, transducing signals received in the root through CLAVATA3/ESR-like (CLE) peptides (MtCLE12 and MtCLE13) with MtCLE12 modified by the hydroxyproline-O-arabinosyl transferase enzyme RDN1 [7, 8] and translocated to the shoot [9] where a receptor complex containing the ortholog of Arabidopsis leucine-rich repeat receptor kinase CLAVATA1, called SUNN in *M. truncatula* [10, 11], the pseudokinase CORYNE (MtCRN), and the receptor-like protein CLAVATA2 (CLV2) [12] receives the signal [13]. Through cytokinin signaling and the transport of *miR2111* to the root [14, 15], the plant is able to limit the number of nodules that form to match the needs of the plant.

Forward and reverse genetics have identified many of the genes involved in this signal transduction pathway and assigned their function either locally (within the root or shoot) or systemically (traveling from root to shoot or shoot to root [4]. In addition, mutations in AON genes such as *SUNN* and *CLV2* orthologs in both legumes and non-legumes, as well as mutations in *RDN1* and disruptions of CLE peptides such as MtCLE53 cause defects in the autoregulation of arbuscular mycorrhizal symbiosis (AOM) [16–19]. Arbuscular mycorrhiza (AM) symbiosis is an intimate relationship between plants and Glomeromycotina fungi, which provide mineral nutrients like phosphorus and N to their host in exchange for carbon [20]. Like AON, AOM negatively regulates AM fungal root colonization, likely to balance symbiotic benefits with carbon investment. Similar to AON, AOM mutants are hypermycorrhizal and display elevated levels of fungal root colonization [16–19], suggesting crosstalk between the two pathways.

Both nodulation and AM symbiosis require nutrient and signal exchange across membranes. In each symbiosis, a microorganism penetrates the plant root and is accommodated in the cells of the plant host. Plant cells involved in both symbioses develop complex membranes with specialized functions for symbiotic exchange of nutrients and molecular communication [21].

In this study, we used forward genetics to identify a root-acting mutation that disrupts both AON and AOM The root-acting *defective in autoregulation* (dar) mutant, which disrupts a membrane protein putatively involved in trafficking or transport of autoregulation signals, causes excess nodulation and increased root colonization by AM fungi**.** Our findings suggest that AON and AOM signals may use a common pathway involving DAR, which is a member of a protein family not previously associated with a role in symbiosis. Phylogenetic analysis reveal that this pathway may be common to other plants, even those that do not undergo either symbiosis.

## Materials and methods

### Plant and genetic resources

The *dar-1* mutant was identified as a hypernodulator from fast neutron-bombarded Jemalong ecotype *Medicago truncatula* by Julia Frugoli during a community screen from which other hypernodulation mutants were also isolated, described in [7]. The *dar1* mutant was backcrossed three times to the wild type Jemalong ecotype A17 by Elise Schnabel to establish the seed stock used in all experiments. The *dar-2* mutant was isolated by Elise Schnabel from the seed pool NF2117 in the R108 ecotype at the Noble Foundation Medicago Mutant Database [22] using computer analysis of flanking sequence tags (FSTs). NF2117 contained an FST corresponding to the *Medtr2g450550/MtrunA17_Chr2g0304631* locus, and a line homozygous for the insertion was isolated by Elise Schnabel from NF2117 progeny. Seeds of both of these backcrossed homozygous lines have been deposited in the Medicago Mutant Database collection.

A vector for rescue, pCDsRed/35S, was constructed from pCAMBIA0390 by replacing a portion of the polylinker with the polylinker region of pCAMBIA3201 (EcoRI to PstI), adding a PCR fragment of 35S from pKANNIBAL [23] between HindIII and BamHI sites using the 35S polylinker replacement primers in Additional file 3 and adding the AscI/HindIII UBQ10pro:DsRed1 fragment of pRedRoot [24]. pCDsRed/35S delivers a T-DNA with a DsRed transformation reporter and constitutive expression of a gene of interest. The full-length CDS (~ 1.6 kb) of *MtrunA17_Chr2g0304631* (*Medtr2g450550* in MtV4.0) was amplified from A17 cDNA, prepared from root RNA with the iScript cDNA Synthesis Kit (Bio-Rad), using primers DAR-F and DAR-R (Additional file 3). The PCR product was ligated into the XhoI and SpeI sites of pCDsRed/35S. The expression construct was selected from a transformation of *E. coli* DH5 Alpha, confirmed by sequencing, and transferred to *Agrobacterium rhizogenes* strain ARqua1 [25] by electroporation for use in hairy root transformation. Expression vectors for *MtCLE12* and *MtCLE13* were as described in [26].

### Nodulation assays

Plant lines were assessed for nodulation phenotype using an aeroponic chamber as previously described [27]. Briefly, following scarification for eight minutes in sulfuric acid and several rinses in water, seeds were placed at 4 °C in the dark for two days. Seeds were allowed to germinate overnight suspended over water on the lid of a petri dish wrapped in foil. One day old seedlings were placed in an aeroponic chamber and grown at 21°C-25°C; 14h/10h light/dark cycle and inoculated as described in [28] allowing continuous misting with nutrient solution lacking N. After three days of growth, plants were inoculated with 150 OD_600_ Units (12 × 10^10^ CFUs) of rhizobia. For nodulation of wild type and mutants in the Jemalong A17 ecotype *Sinorhizobium/Ensifer medicae* ABS7 was used [29], and for wild type and mutants in the R108 ecotype, *Sinorhizobium/Ensifer* Rm41 was used [30]. Cultures (100 ml) were grown in liquid TY medium shaking at 30 °C, collected by centrifugation at 2500 × g for 10 min and resuspended in nutrient medium. Nodule counts were performed after 10 to 14 days.

### Mycorrhizal assays

Seeds of *M. truncatula* ecotype A17 and mutant lines derived from A17 (*sunn-4* and *dar-1)* were scarified using sandpaper to break the seed coat and surface-sterilized with 70% ethanol for 7 min. The seeds were then carefully washed 3–4 times in sterile water before being placed on a rotator to incubate for around 2 h at room temperature. Imbibed seeds were incubated overnight at 4°C and germinated at room temperature in a petri dish with moist, sterilized filter paper for 4 days. Seedlings were transplanted to 20.5 cm long cones containing a 2:1 mixture of washed and autoclaved fine sand and vermiculite. Each cone carrying a single plant received 250 *Rhizophagus irregularis* spores (Premier Tech, Canada) that were placed 5 cm below the top of the cones [16]. Plants were grown in a growth chamber with a 16-h light cycle (24°C) and an 8-h dark cycle (22°C). Each *M. truncatula* plant was fertilized with modified 15ml half-strength Hoagland's fertilizer every 4th day, which contained 20 µM phosphate.

To visualize AM fungal colonization, at 4 weeks post-planting *M. truncatula* roots were stained with 0.2 mg/ml WGA-Alexafluor 488 (Invitrogen) [16] and observed using a Leica Thunder Imager (Leica M205 FA). Total root length colonization was evaluated using the grid line method [31]. R software was used for the box plot and statistical analysis (multiple comparisons were analyzed by ANOVA and Tukey’s HSD post-hoc test).

### Grafting and rescue experiments

Shoot to root grafting was performed on *M. truncatula* seedlings as previously described [32]. Grafted plants were transferred to perlite pots and watered for several days with dilute complete fertilizer. Once the transferred plants had begun to grow, the pots were rinsed with water and watered from then on with nutrient solution lacking N. After five days, the N starved plants were inoculated with 150 OD_600_ Units (12 × 10^10^ CFUs) of *S. medicae* ABS7 in 6 ml of nutrient solution. Nodule counts were performed at 14 days after inoculation.

Roots expressing transgenes were generated via *Agrobacterium rhizogenes*-mediated hairy root production as described in [24] using strain ARqua1 [25] containing the appropriate binary vector. Plants were grown on 10 cm square plates until sufficient root growth had occurred; non-transgenic roots were then removed, and plants were transferred to perlite pots and assayed for nodulation as described above.

### Mapping of the dar locus

To determine the chromosomal location of the *dar* lesion, *dar-1* was crossed with polymorphic ecotype A20, and hypernodulating F2 progeny were evaluated with known Cleaved Amplified Polymorphic Sequence (CAPS) and length markers from across the genome [33]. Tight association was found between hypernodulation and markers PFK and PGK I from the middle of chromosome 2 (positions 9 Mb and 34 Mb in *M. truncatula* genome v5), with 63% and 84% of 115 individuals homozygous for the allele from the *dar-1* parent at PFK and PGK I [33], respectively. Additional markers were assessed between these locations. Using a length polymorphism assayed using ES50020 primers (Additional file 3) at 22 Mb and an Alu I polymorphism assayed using Medtr2g050180-C and D primers (Additional file 3) at 28 Mb, we found tight linkage, with over 98% of individuals homozygous for the allele from the *dar-1* parent at each locus. The region flanked by these markers contains only one annotated gene. A series of PCR primers from across this 6 Mb region (Additional file 4) detected a deletion of between 8 and 17 kb that included the entire *MtrunA17_Chr2g0304631* gene, and no other annotated genes.

### Sequencing of AON genes in dar-1

Other AON genes in the *dar1* mutant line were analyzed by Sanger sequencing of PCR products generated from genomic DNA with the primers used for sequencing in the initial cloning of *SUNN* [11], *RDN1* [7] and *CRN* [12]. All sequencing in this manuscript was performed by the Genomics Facility at Arizona State University and aligned with Sequencher software (Gene Codes Corporation).

### Computational methods and statistical analyses

The phylogenetic tree in Fig. 8 and Additional file 2 were constructed from sequences indicated in the trees using MEGA X with default settings [34]. Clustal Omega [34] was used to create the alignment in Additional file 2. DAR protein topology was initially predicted using psort [35] and Phobius [36], then drawn freehand in PowerPoint. Initially, we created an AlphaFold structure by manually uploading the full-length protein sequence in https://alphafoldserver.com/ (created by Google Deepmind). However, the same Alphafold structure was updated in the UniProt database that uses the same AI system created by Google Deepmind. Thus, the DAR protein structure with confidence scores as well as graph showing expected position error of residues was extracted from the UniProt database with protein ID A0A072VIE1.

For statistical analysis of multiple treatment groups, we first checked the data for normality using the Shapiro–Wilk test. For the data that did not follow a normal distribution (Shapiro–Wilk *p*-value < 0.05), we performed the Kruskal–Wallis test for statistical analysis followed by Dunn's post-hoc test for pairwise comparisons, incorporating Bonferroni correction. For normally distributed data (Shapiro–Wilk p-value ≥ 0.05), we conducted ANOVA (confidence interval of 95 percentile) to compare means, followed by Tukey's HSD post-hoc analysis.

## Results

### Isolation of hypernodulation mutant *dar-1*

**Fig. 1:**
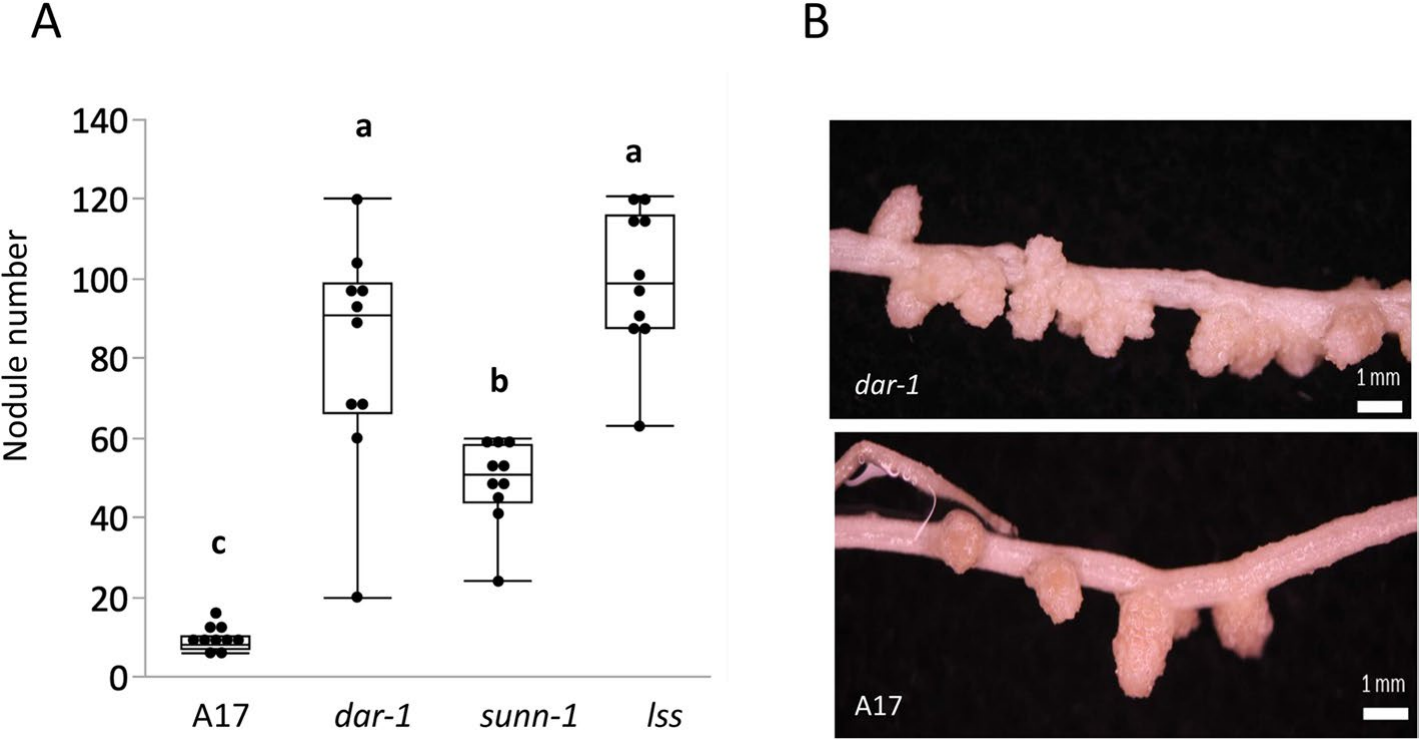
The *dar-1* mutant has a hypernodulation phenotype. **A** Nodule counts at 10 dpi for *dar-1* compared to wild type A17 and hypernodulating mutants *sunn-1* and *lss*. *dar1* and *lss* display higher nodule numbers than wildtype and the hypernodulating *sunn-1* mutant (Kruskal–Wallis test, *p* = 6.613 × 10^–07^; different letters denote significant differences (*p* < 0.05) in pairwise comparisons using Dunn’s post-hoc test; *n* = 10 plants per genotype.) **B** Nodules at 18 dpi in *dar-1* (upper panel) and A17 (lower panel)

Screening of a fast neutron bombardment mutagenized population of *Medicago truncatula* led to the isolation of a hypernodulating mutant line designated *defective in autoregulation* (*dar-1*). No mutations were found in the sequences of AON genes *SUNN*, *CRN*, or *RDN1* in *dar-1*. The mutant produced approximately tenfold more nodules than wild type (Fig. 1A). This is higher than observed in the original *M. truncatula* AON mutant *sunn-1* but is similar to the level found in *lss*, an AON mutant with low *SUNN* expression and previously observed in the *sunn-4* loss of function mutant [27]. The nodules produced by *dar-1* are similar to wild type nodules at 14 days after inoculation (Fig. 1B).

### DAR acts from the roots

**Fig. 2:**
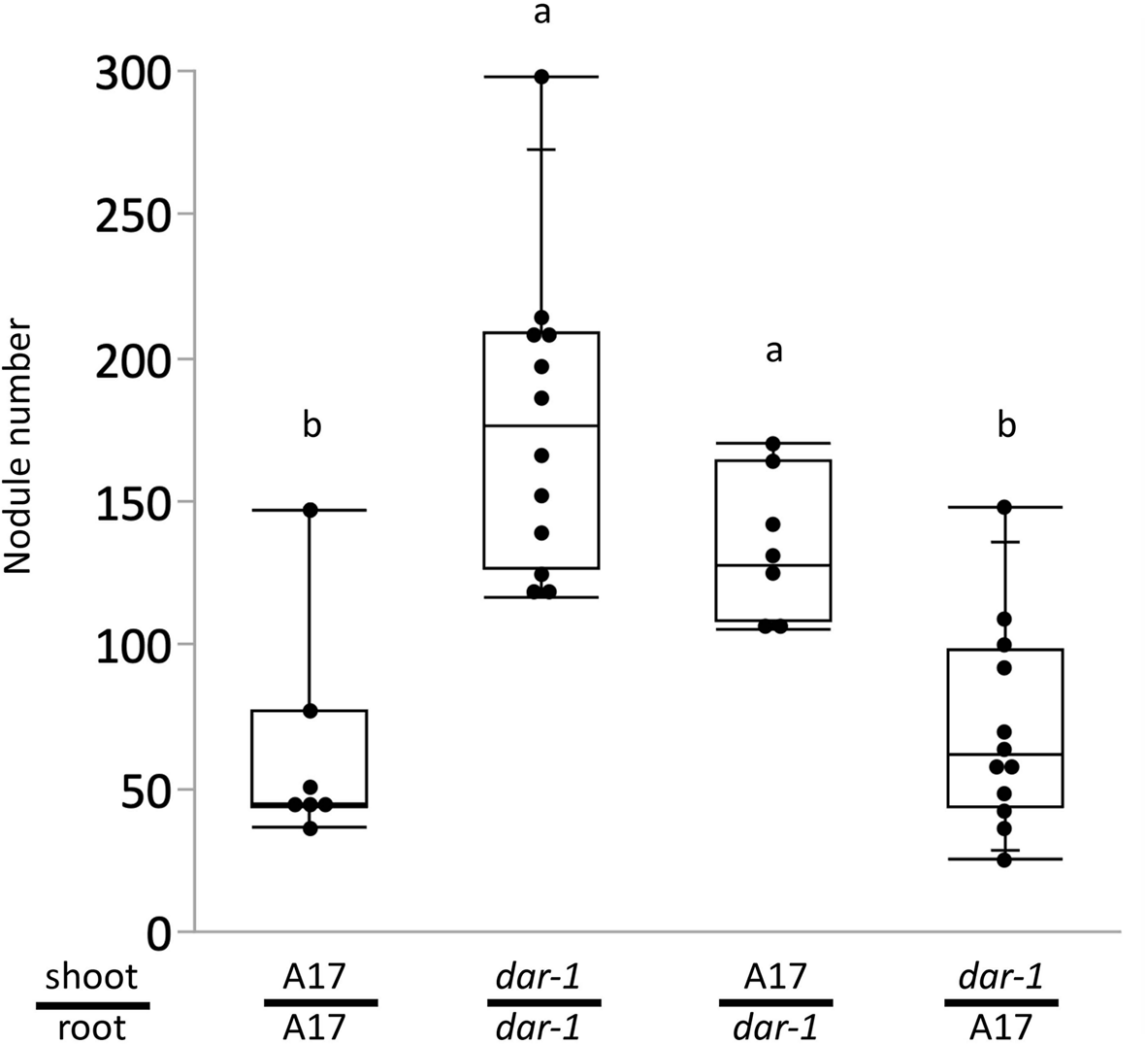
*dar-1* acts in the root to control nodule number. Nodule counts on grafted plants 21 days after inoculation with *Sinorhizobium medicae* ABS7. Grafted plants with *dar-1* roots nodulated significantly more than wild type self-grafts, while plants with *dar-1* only in the shoots were similar to wild type (ANOVA *p* = 3.28 × 10^–07^, different letters denote significant differences (*p* < 0.05) in pairwise comparisons using Tukey’s HSD test; *n* = 7 to 12 plants per grafting combination)

As mentioned above, the AON pathway involves long distance signaling between roots and shoots. Some AON pathway genes, like *MtCLE12*, *MtCLE13* and *MtRDN1*, act in the roots to control nodule number, while others, like *SUNN* and *MtCRN*, act in the shoots. We performed reciprocal grafting between *dar-1* and wild type to determine if the impact of the *dar-1* lesion on nodule number was from the roots or from the shoots (Fig. 2). Self-grafts of wild type and *dar-1* recapitulated the phenotypes of the original lines. However, plants composed of wild type shoots and *dar-1* roots exhibited hypernodulation, while plants composed of *dar-1* shoots and wild type roots nodulated at a wild-type level. This demonstrates that the impact of the *dar-1* lesion on nodule number control is from the roots.

### DAR is a member of the AON pathway

**Fig. 3:**
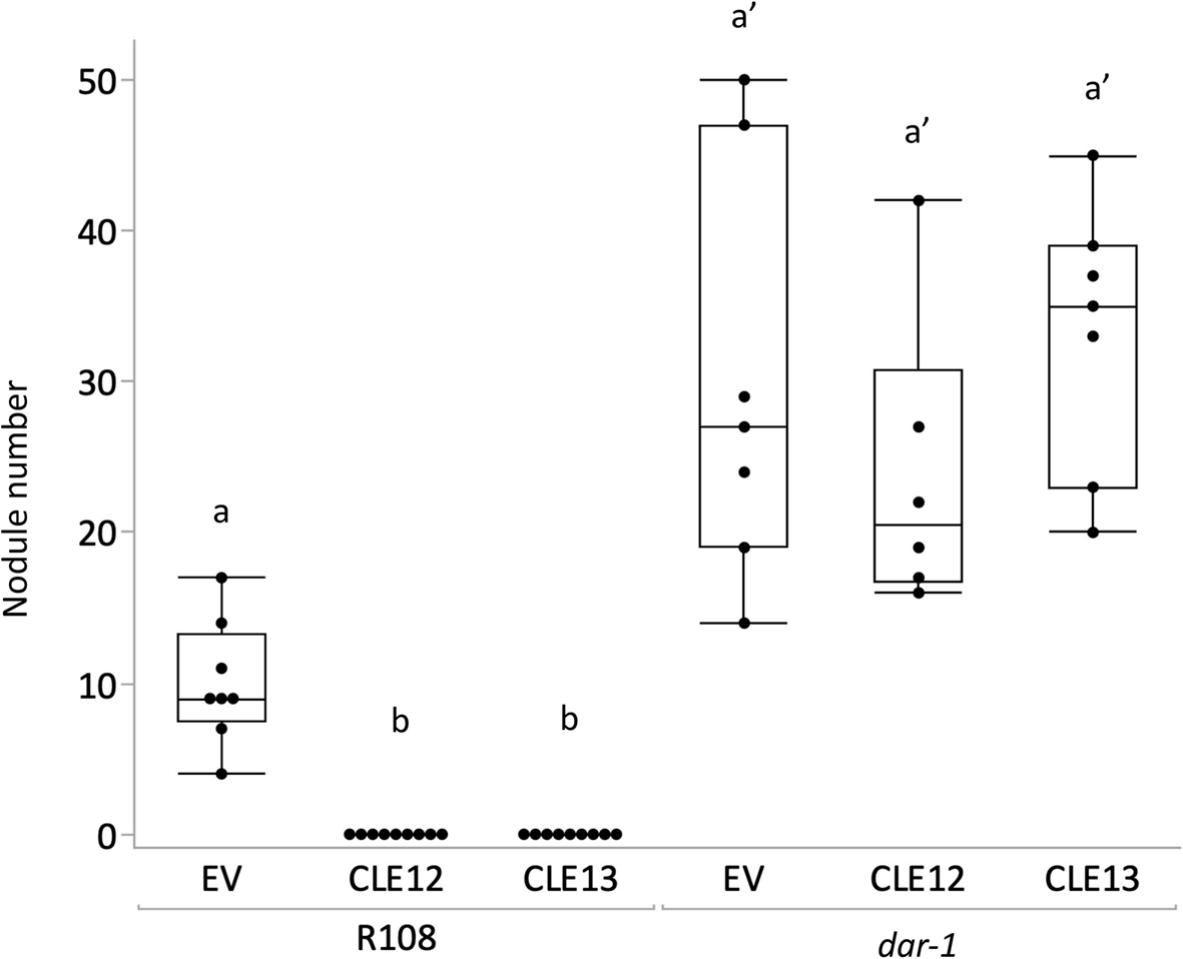
*dar-1* is not suppressed by CLE peptide expression. Wild type R108 and *dar-1* plants with roots carrying control (EV), p35S::*CLE12*, or p35S::*CLE13* plasmids were inoculated with *Sinorhizobium medicae* and assessed for nodulation after 21 days *(n* = 7 to 9 plants per condition). Nodulation was suppressed by *CLE12* or *CLE13* expression in the wild type (Kruskall-Wallis test *p* < 0.05) but not in *dar-1* (Kruskal–Wallis test *p* > 0.05). Different letters denote significant differences (*p* < 0.05) in pairwise comparisons for each genotype using Dunn’s post-hoc test

The early stages of AON involve the induction of CLE peptides genes *MtCLE12* and *MtCLE13* [37]. While ectopic expression of either of these peptides in wild type roots suppresses nodulation, in *sunn* mutants, hypernodulation persists in the presence of CLE peptide expression, suggesting that SUNN is downstream of the CLE peptides in the AON pathway [13]. To determine if *dar-1* is downstream of the CLE peptides in the AON pathway, we expressed *MtCLE12* and *MtCLE13* in *dar-1* roots as in [26] and looked for an impact on nodule number (Fig. 3). In wild type, the expression of either *MtCLE12* or *MtCLE13* abolished nodulation. Conversely, *dar-1* roots expressing either of the CLE peptide genes remained hypernodulated, suggesting that the *dar-1* lesion disrupts the AON pathway downstream of the CLE peptides.

### Identification of the causative gene for the observed AON defect

**Fig. 4:**
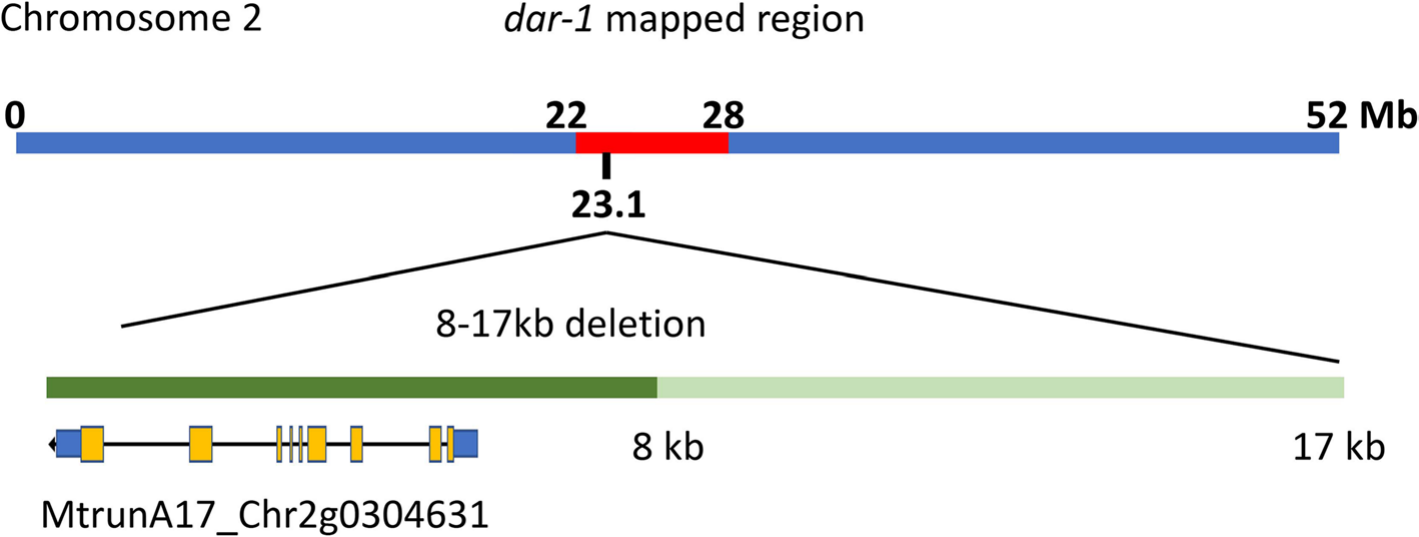
Identification of a gene deletion in a region associated with the *dar-1* phenotype. Analysis of progeny of a mapping cross between the *dar-1* mutant and ecotype A20 revealed linkage of the *dar-1* lesion to a 6 Mb region between nucleotide positions 22 and 28 Mb on chromosome 2 (in red). A deletion of between 8 and 17 kb was identified in *dar-1* near nucleotide position 23.1 Mb which included a single gene, *MtrunA17_Chr2g0304631*

To identify the locus in *dar-1* responsible for the AON phenotype, we generated a polymorphic mapping population from a cross with *M. truncatula* ecotype A20. The F2 progeny segregated hypernodulation mutants at a 3:1 ratio (Table 1), suggesting a single recessive locus was responsible for the phenotype but the Chi-Square value was non-significant. Co-segregation of *dar-1* genotype (A17) markers with the hypernodulation phenotype in the F2 progeny was observed for the central region of chromosome 2 (Fig. 4) and this close association with the centromere is likely responsible for the slightly skewed segregation observed. We delineated the lesion to a region between chromosomal positions at 22 and 28 Mb (genome version MtrunA17r5.0). Within this region we discovered a deletion of 8 to 17 kb at chromosomal position 23.1 Mb that contained a single predicted gene, *MtrunA17_Chr2g0304631*.

**Table 1.**
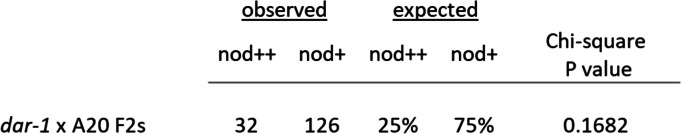
*dar* behaves like a single recessive locus

**Fig. 5:**
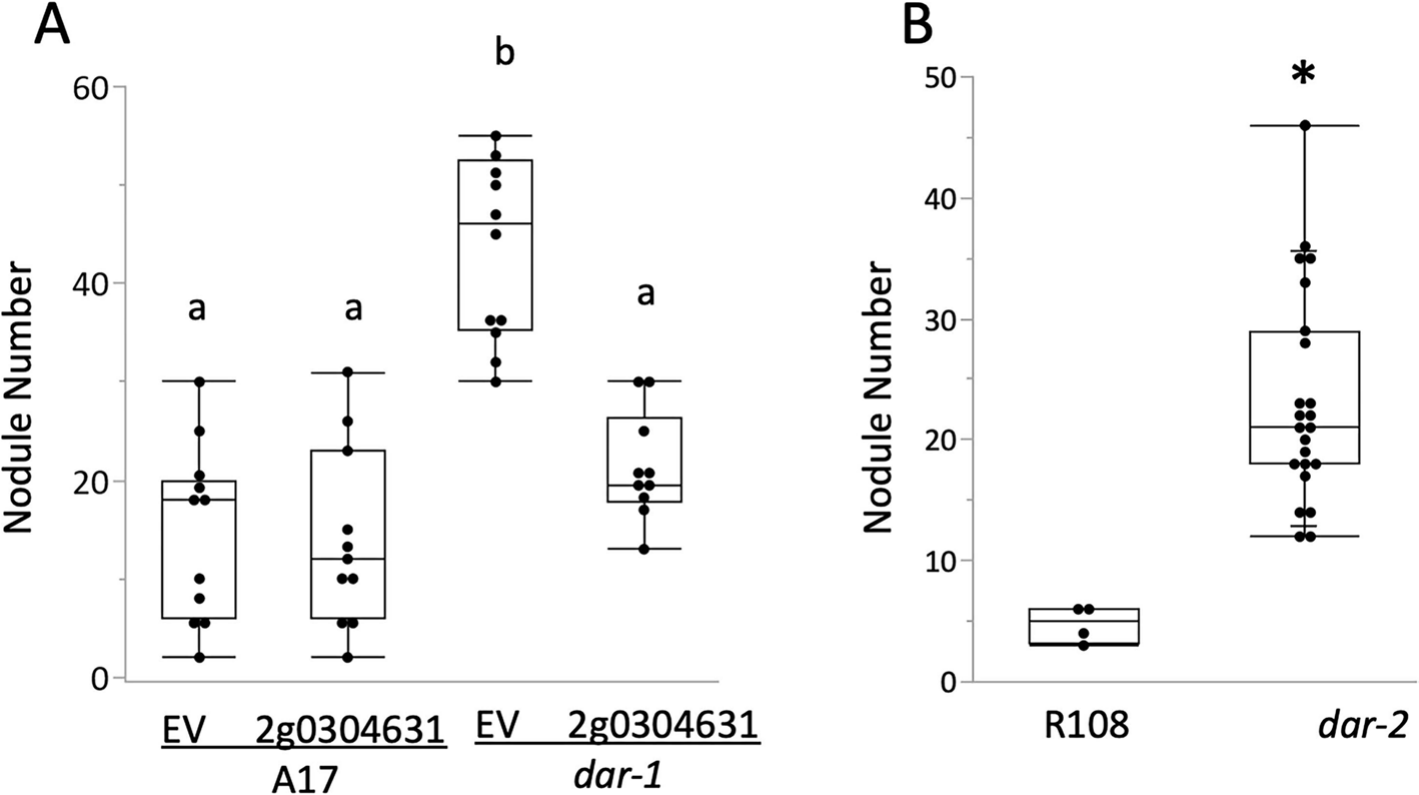
Rescue of *dar-1* and identification of *dar-2* (**A**) Ectopic expression of *MtrunA17_Chr2g0304631* in roots of the *dar-1* mutant restores nodule number regulation. The *dar-1* plants with the empty vector were significantly different from wild type, while *dar-1* plants with the *MtrunA17_Chr2g0304631* expression construct were similar to wild type (Kruskal–Wallis test *p* = 4.799 × 10^–06^; different letters denote significant differences between samples (*p* < 0.05) in pairwise comparisons using Dunn’s post-hoc test). For each condition, 10 to 11 plants were analyzed. **B** A line derived from NF2117 (*dar-2*) carrying a *Tnt1* insertion in *MtrunA17_Chr2g0304631* showed an increased number of nodules compared to its parental wild type line, R108. (*, *p* < 5 × 10^–4^, Student’s t-test; *n* = 4 to 23 plants)

To test whether the deletion of *MtrunA17_Chr2g0304631* was responsible for the AON phenotype in *dar-1*, we introduced the coding sequence of the gene under the control of the *CaMV35S* promoter into plant roots via *Agrobacterium rhizogenes*-mediated transformation (Fig. 5A). The expression of the gene in wild type roots caused no change in nodulation levels, but in *dar-1* roots, expression of *Mtrun17_Chr2g0304631* resulted in a reduction in nodulation to wild type levels, rescuing the mutant phenotype. We also isolated a mutant line derived from a pool of *Tnt1* insertions in the R108 genotype [38] carrying a homozygous insertion within *MtrunA17_Chr2g0304631*. Nodulation in the *Tnt-1* insertion mutant was approximately sixfold higher than in wild type (Fig. 5B), and this allele was designated *dar-2*. The results support the conclusion that *MtrunA17_Chr2g0304631* is involved in AON and that the disruption of this gene is responsible for the observed hypernodulation phenotypes in *dar-1* and *dar-2*.

### *dar-1* mutants are defective in AM symbiosis regulation

**Fig. 6:**
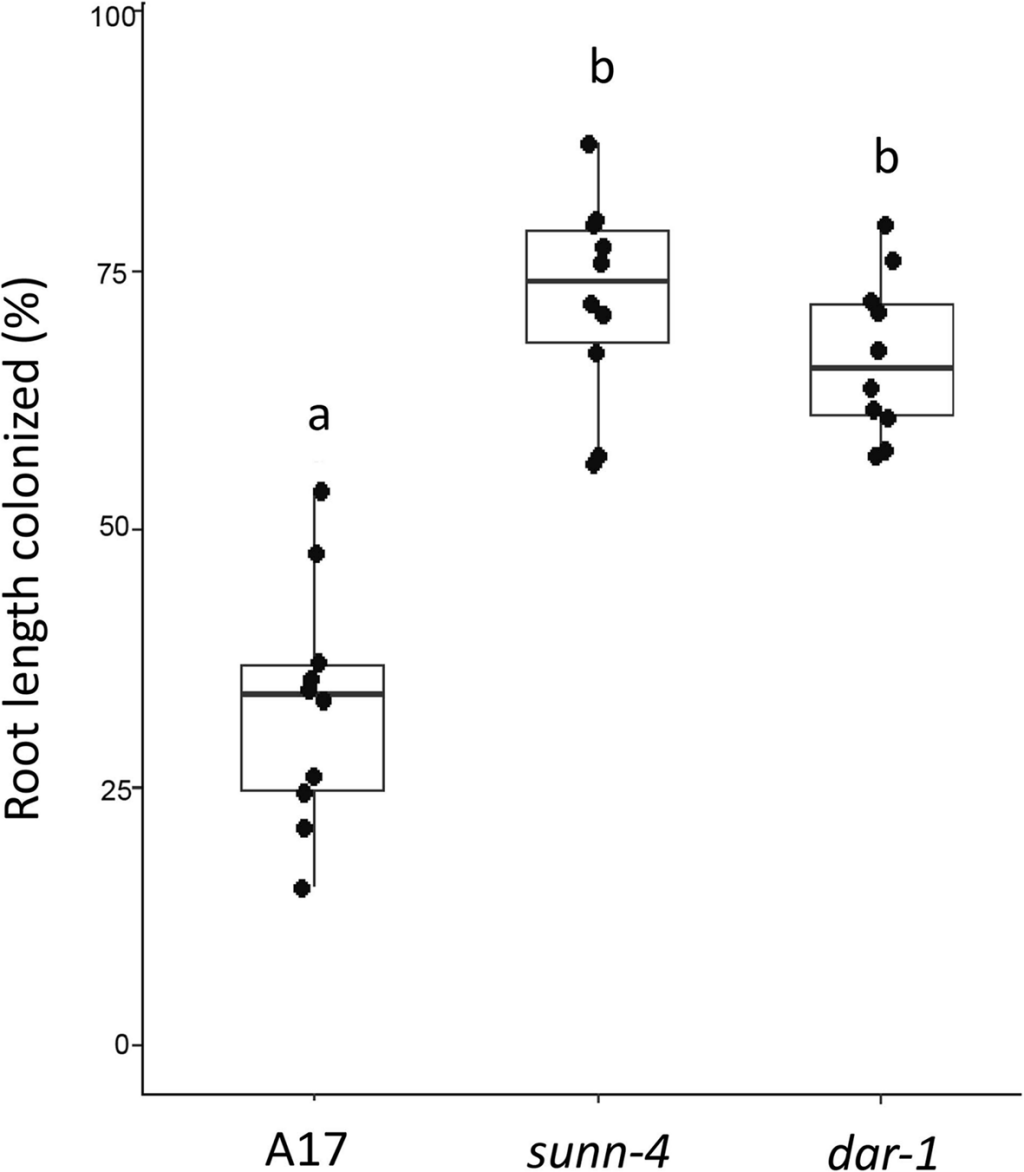
*dar-1* mutants show elevated root length colonization with the AM fungus *Rhizophagus irregularis* relative to the A17 wildtype. Colonization levels in *dar-1* and *sunn-4* mutants were indistinguishable. For each line, 10 colonized root systems were analyzed. Statistical analysis was performed using Kruskal–Wallis test followed by Dunn’s test for pairwise comparisons. Different letters denote significant differences (*p* < 0.05)

Because the *sunn-4* mutant had previously displayed autoregulation defects in arbuscular mycorrhizal symbiosis as well as N-fixing symbiosis [16], we wanted to test if *dar-1* mutants displayed a similar defect. We assessed overall root length colonization of *R. irregularis*-colonized roots (10 biological replicates per line). The percentage of root length colonized in *dar-1* mutants was significantly elevated compared to A17 and resembled the hypermycorrhizal phenotype in *sunn-4* mutants, suggesting *DAR* is a negative regulator of AM symbiosis, just like *SUNN* (Fig. 6).

### Expression of *DAR* during symbiotic interactions

**Fig. 7:**
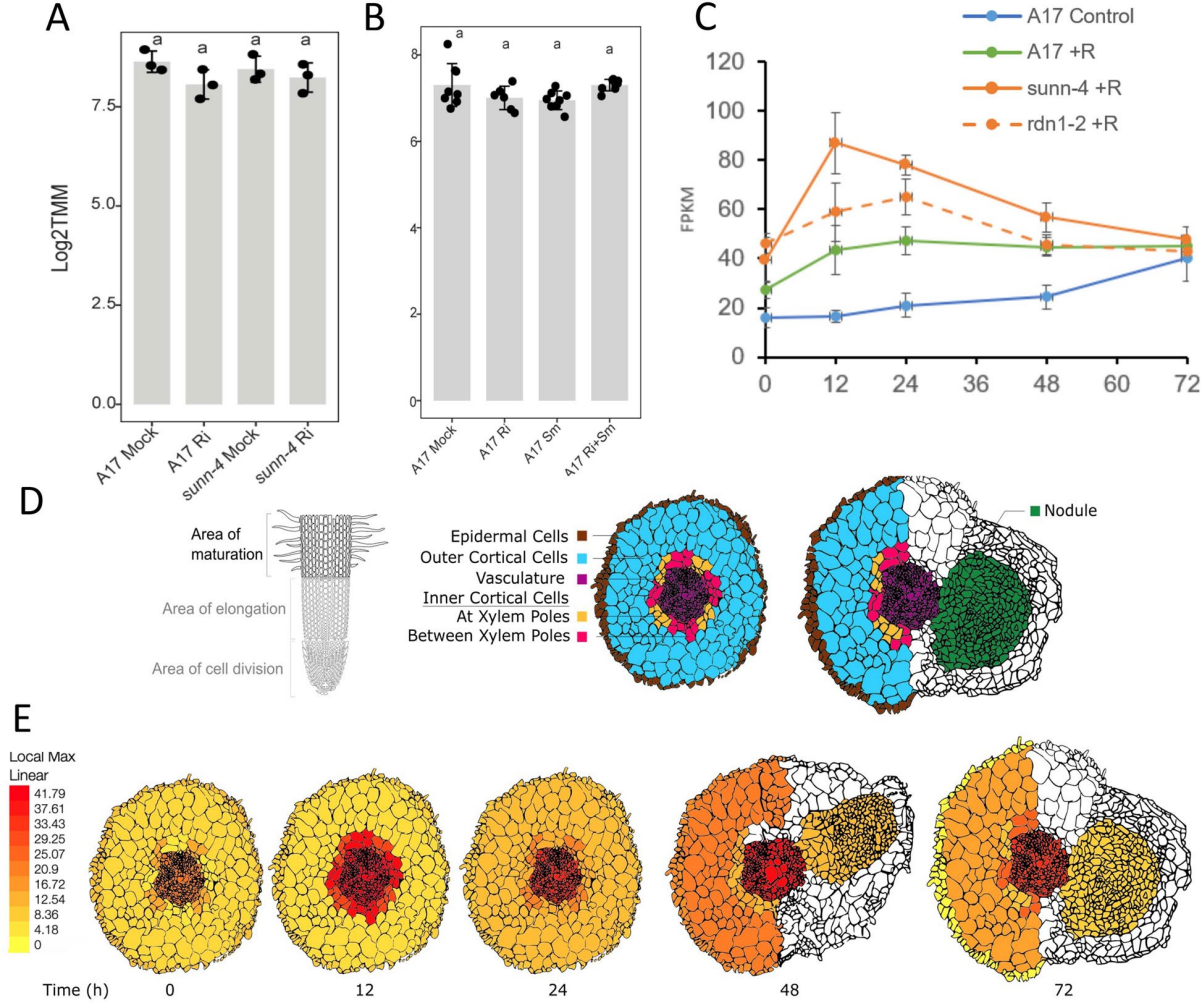
Expression of *DAR1* in *M. truncatula* root tissues in response to inoculation with arbuscular mycorrhiza and rhizobia. **A** Normalized expression (Log2TMM) of *DAR* expression in mock- vs *R. irregularis*-inoculated roots in A17 or *sunn-4* roots harvested 21 post days inoculation. Data from [17]. **B** Normalized expression (Log2TMM) of *DAR* expression in roots inoculated with *Rhizophagus irregularis* (*Ri*) and/or *Sinorhizobium meliloti* (*Sm*) individually or in combination; roots harvested 7 weeks post inoculation. Data from [43]. ANOVA was used to compare means, followed by Tukey's HSD post-hoc analysis. **C** Expression of *DAR* normalized by Fragments Per Kilobase of transcript per Million mapped reads (FPKM) in roots over 72 h post inoculation with *Sinorhizobium medicae* (+ R) in A17, *sunn-4*, and *rdn1-2.* Data from [44]. **D** Schema for displaying tissue expression using default parameters at https://bar.utoronto.ca/eplant_medicago/. Data are compiled from three independent replicates and colors are painted on an entire tissue regardless of which cells within that tissue expressed the gene. **E** Relative expression of *DAR* in each tissue at indicated hours post inoculation

Examination of public data sets for expression of *DAR* in roots undergoing arbuscular mycorrhizal interactions [17] (Fig. 7A and [16]), mycorrhizal plus rhizobial interactions [39] (Fig. 7B) and rhizobial interactions alone [40] (Fig. 7C) showed the *DAR* gene is not significantly differentially regulated during either symbiosis. The tissue level expression of *DAR* in roots responding to rhizobia during the first 72 h post-inoculation was obtained from the ePlant resource for early nodulation [41] and displayed in Fig. 7D and Fig. 7E. In this data set, *DAR* is expressed mainly in the vasculature and inner cortical cells, with little change in expression level over time.

In a recent single-nucleus transcriptomic assay, *DAR* transcripts were specifically detected in the phloem cell cluster in *M. truncatula* roots colonized by the AM fungus *R. irregularis* [42]. Single-nucleus transcriptomic analysis of *M. truncatula* roots responding to rhizobia over time also revealed *DAR* expression was concentrated in the stele cluster with markers for phloem, and at 96 h post inoculation *DAR* transcripts began to appear in the nodule primordia and in a cluster annotated as the stele responding to the infection [43]. Localization of the transcript to stele/phloem was also confirmed in the single time point (48 h post inoculation) dataset of [44] and in a single nucleus dataset generated by inoculation with nod factor versus live rhizobia [45], while lower expression can be observed in some of the other cell types.

### *DAR* encodes a membrane protein with putative roles in cellular transport or trafficking

**Fig. 8:**
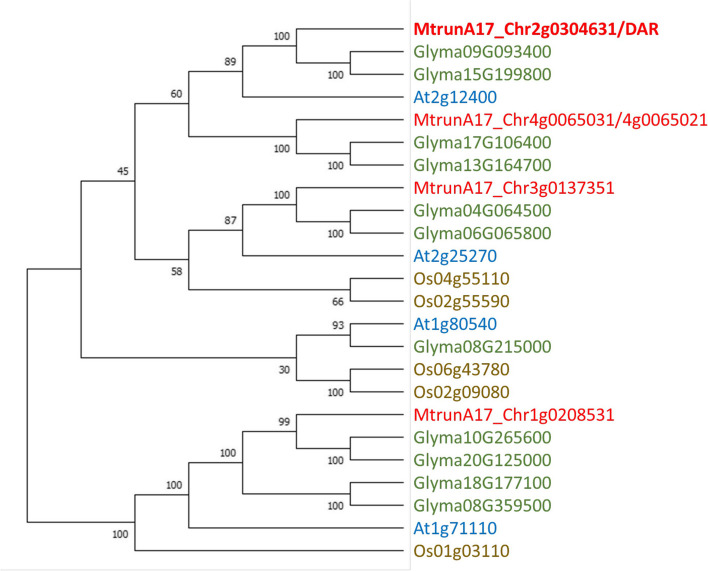
DAR1 related proteins in *M. truncatula*, Arabidopsis, rice, and soybean. A Neighbor-Joining tree of DAR and related proteins from *Medicago truncatula*, *Glycine max*, *Arabidopsis thaliana*, and *Oryza sativa*. The bootstrap consensus tree was inferred from 500 replicates. The percentage of replicate trees in which the associated taxa clustered together in the bootstrap test shown next to the branches. A family member in *M. truncatula* annotated as *Medtr4g116290* in v4.1 was split into two transcripts in v5.0 (*MtrunA17_Chr4g0065031* and *MtrunA17_Chr4g0065021*); the coding sequence predicted from the v4.1 transcript was used for this analysis

There are three proteins annotated in the *M. truncatula* genome with identity to DAR of over 40% and two more distantly related proteins (20 to 23% identity), all annotated as “putative transmembrane proteins.” Alignment of *M. truncatula* and Arabidopsis sequences (Additional file 1) shows sequence similarity across the length of the proteins, including within the transmembrane domains. DAR-related proteins are also present in the genomes of other plants, including microalgae. The relationship between DAR and closely related proteins from *M. truncatula*, Arabidopsis, rice, and soybean are shown in Fig. 8. A broader view of the protein family is shown in Additional file 2, including all family members from *M. truncatula* compared to *Arabidopsis thaliana*, *Oryza sativa*, *Glycine max*, *Physcomitrella patens*, *Selaginella moellendorffii*, *Micromonas commode* RCC299, and *Ostreococcus lucimarinus*.

**Fig. 9:**
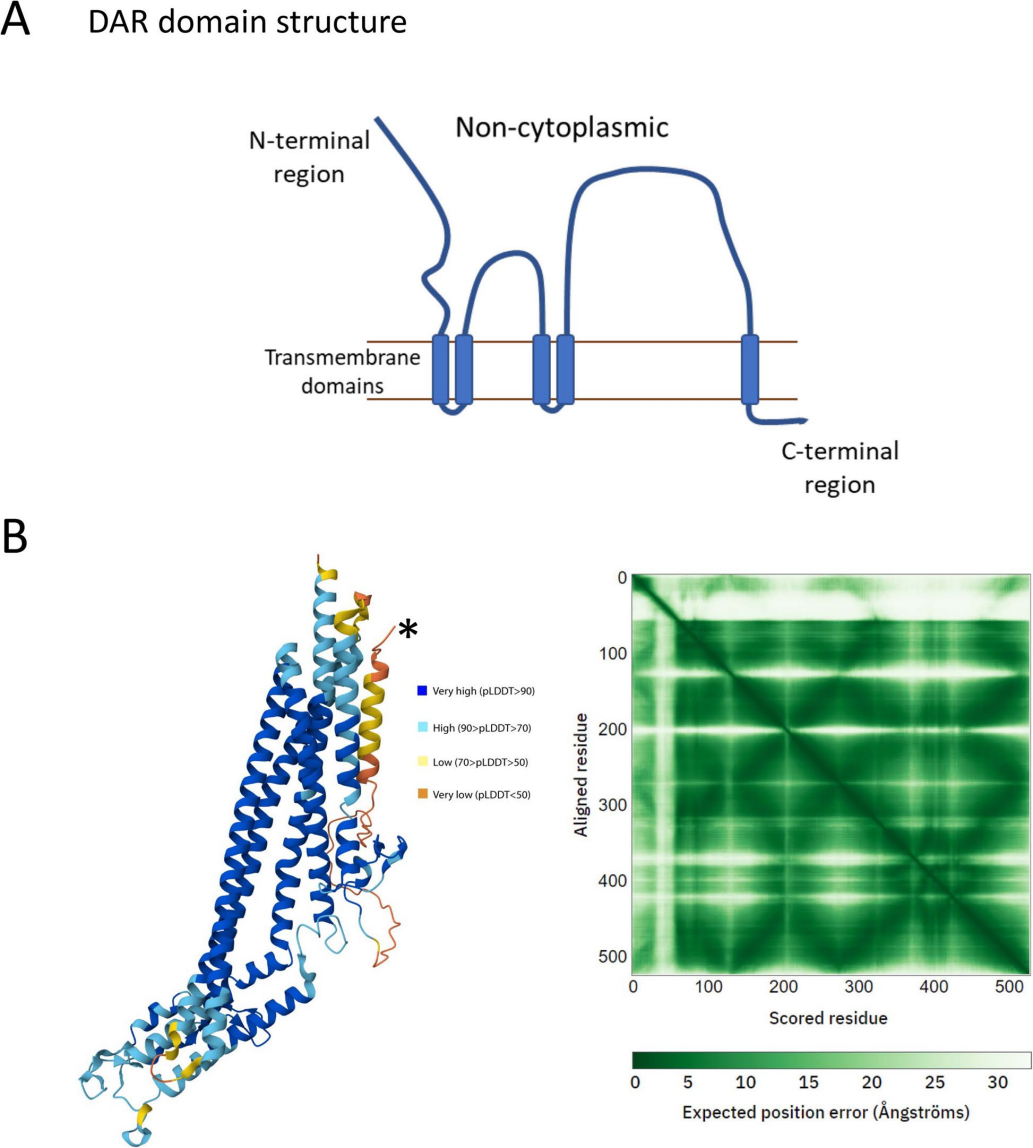
Protein structure of DAR (**A**) The transmembrane topology of DAR. The predicted structure includes five transmembrane spanning domains. Cytoplasmic links between consecutive TM domains are short, while on the side of the membrane annotated as non-cytoplasmic by Phytozome there are regions of 78 to 185 amino acids. **B** Displayed is the AlphaFold predicted structure of the DAR protein and corresponding confidence metrics. The left panel displays the predicted 3D structure of the DAR protein with the respective confidence level for each region of the DAR. * indicates the start of the protein. Blue: Very high confidence (pLDDT > 90), Cyan: Confident (90 > pLDDT > 70), Yellow: Low confidence (70 > pLDDT > 50), Orange: Very low confidence (pLDDT < 50). The right panel shows the predicted alignment error (PAE) heatmap that represents the expected position error (in Ångströms) for each pair of residues in the predicted structure, where darker and lighter green indicate the lower and higher expected error rate respectively. The diagonal represents self-alignment (perfect prediction). The color scale at the bottom shows the range of expected position errors

Phobius structural predication indicates DAR is a predicted 528 amino acid type III membrane protein with a cleavable signal sequence and five transmembrane spanning domains (Fig. 9 A). The majority of the protein is predicted to be located on the non-cytoplasmic side of the membrane, with only short loops between transmembrane domains and a 15 amino acid C-terminal tail facing the cytoplasm. AlphaFold analysis (Fig. 9B) predicts multiple helices with very high to high confidence, while the N terminus of the protein, especially the signal peptide, has a low confidence structure.

Although the molecular function of DAR remains elusive, two closely related proteins from Arabidopsis (At2g12400 and At1g71110 in Fig. 8) were found in a yeast two hybrid screen of membrane and signaling proteins [46]. A search of the Membrane-based Interactome Database [47] for interacting partners for each of these proteins revealed multiple interactions for both (At2g12400, *n* = 19; At1g71110, *n* = 18) with eleven proteins in common, including Bet1-Like SNARE 1–2. Analysis of the localization of the Arabidopsis interacting partners at the Subcellular Localization Database for Arabidopsis Proteins [48] determined a consensus localization of the proteins (SUBcon), with many assigned to the golgi, plasma membrane, or endoplasmic reticulum (Table 2). In line with a potential function in cellular transport or membrane trafficking, DAR is associated with the GO terms ‘plasma membrane’ (GO:0005886), ‘plasmodesma’ (GO:0009506), and ‘membrane’ (GO:0016020).

**Table 2.**
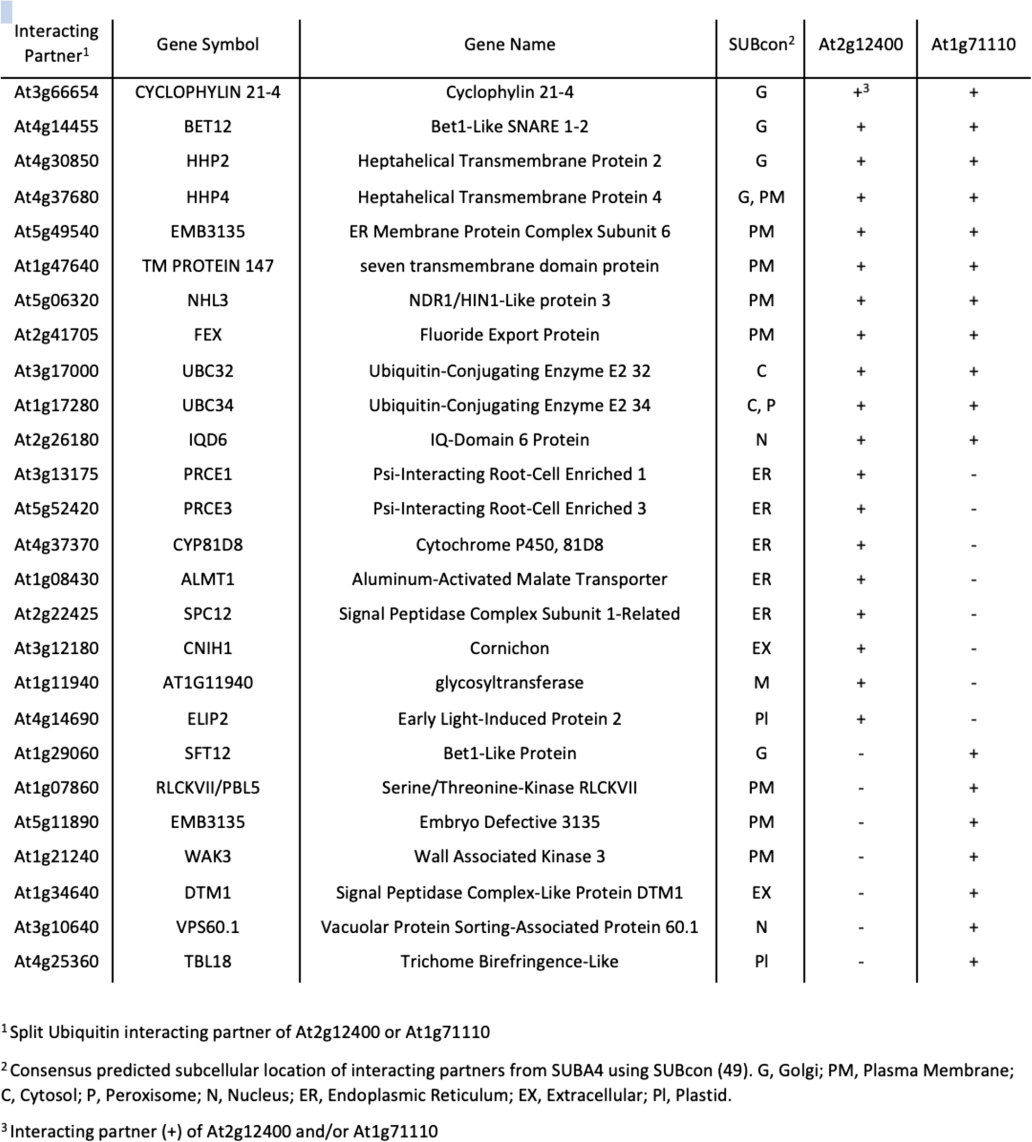
Interacting partners of DAR-related proteins in Arabidopsis, At2g12400 and At1g71110

## Discussion

The phenotype of plants with mutations in *DAR* clearly shows the involvement of the protein in both AOM and AON. While the two alleles of *dar* vary in nodule number phenotype, they both hypernodulate compared to the wild type controls. The variation in nodule number could be a function of the molecular structure of the gene disruption (the Jemalong A17 allele is a complete deletion and the R108 allele is a T-DNA insertion into the gene), a more likely explanation is the ecotypes themselves. The Jemalong A17 ecotype and the R108 ecotype are genetically different [49] and have differences in responses to minerals, drought, and disease resistance [50–53]. Under our growth conditions they also have different wild type nodule numbers (Fig. 1 and Fig. 5) and are inoculated with different rhizobial strains; this likely accounts for the variation in hypernodulation phenotype. Based on the mutant phenotypes, DAR is a protein that performs a function required for AON and AOM. Because the gene is conserved in Arabidopsis, which forms neither symbiosis, and because *DAR* is expressed in plants not interacting with symbionts, we hypothesize that DAR has additional roles independent of symbiotic interactions.

The symbiotic interface for both arbuscular mycorrhizal symbiosis and nodulation is created in root cells upon infection by the microbial symbiont. Upon symbiosis establishment, long-distance signals that negatively regulate nodulation or mycorrhizal colonization, travel through the vasculature (reviewed in the introduction). While DAR plays a role in *M. truncatula* symbioses as determined by phenotypic analysis, it is not specific to symbiosis, being expressed in both the presence and absence of a symbiont (Fig. 7). However, its orthologs in Arabidopsis associate with Bet1-Like SNARE 1–2 and 10 other proteins in common, many of which localize to the golgi, plasma membrane, or endoplasmic reticulum (Table 2). Combined with single nuclei transcriptomics data that suggest vasculature/phloem localization, these data suggest DAR could be involved in cargo movement into the vasculature.

Since DAR appears to be involved in the SUNN-CLE pathway (Fig. 3), and its structure indicates a role in membrane transport (Figs. 8 and 9), we propose that DAR is involved in transporting cargo in the root vasculature or regulating transport, and we speculate that one possible cargo may be a signal involved in AON and AOM. The signal could be CLE peptides (which are involved in both AON and AOM), miRNAs (also involved in both symbioses), or another signal that is not yet determined. DAR could also be involved in regulating auxin transport, long known to influence nodule number [54] and AM symbiosis [55]. Localization of PIN auxin efflux carriers and AUX/LAX influx carriers requires directional vesicle transport, and both transporter classes have been shown to affect nodule number [56–59]. Overall, the identification of mutations in *DAR* now allows further experimentation to determine the cellular function of DAR protein and connect it to the signal transduction pathways of AON and AOM.

## Conclusions

Here, we report the identification of *M. truncatula* DAR as a new root-acting protein involved in symbiosis autoregulation, with roles in both AON and AOM. *DAR* encodes a member of protein family associated with membranes but not previously implicated in symbiosis regulation. We hypothesize that DAR may be involved in the transport or trafficking of autoregulatory signals, potentially the root-derived CLE peptides or miRNAs.

## Supplementary Information

Supplementary Material 1.

Supplementary Material 2.

Supplementary Material 3.

Supplementary Material 4.

## Data Availability

The data generated and/or analyzed during the current study are available in the following repositories: Genomic, cDNA and protein sequences of DAR are available at the *M. truncatula *version 5 genome browser (https://medicago.toulouse.inra.fr/MtrunA17r5.0-ANR/) under gene ID MtrunA17_Chr2g0304631. Expression data is available at the ePlant Expression Browser (https://bar.utoronto.ca/eplant_medicago/)    under gene ID Medtr2g450550. Seed for *dar-1* and *dar-2* mutants, as well as mutant and wild type lines used as controls in this study are available at the *Medicago *Mutant Database (https://medicago-mutant.dasnr.okstate.edu/mutant/index.php).
