# Correction: The Defective in Autoregulation (DAR) gene of Medicago truncatula encodes a protein involved in regulating nodulation and arbuscular mycorrhiza

**DOI:** 10.1186/s12870-025-06215-4

**Published:** 2025-02-14

**Authors:** Elise Schnabel, Sagar Bashyal, Cameron Corbett, Tessema Kassaw, Stephen Nowak, Ramsés Alejandro Rosales-García, Rooksana E. Noorai, Lena Maria Müller, Julia Frugoli

**Affiliations:** 1 Department of Genetics and Biochemistry, Clemson University, Clemson, SC 29634 USA; 2 Department of Biology, University of Miami, Coral Gables, FL 33124 USA; 3 Department of Biological Sciences, Clemson University, Clemson, SC 29634 USA; 4 Clemson University Genomics and Bioinformatics Facility, Clemson University, Clemson, SC 29634 USA; 5 Plant Molecular and Cellular Biology Laboratory, Salk Institute for Biological Studies, La Jolla, CA 92037 USA; 6 School of Biological Sciences, University of California San Diego, San Diego, CA 92093 USA; 7 Department of Biology, West Virginia Uni- versity, Morgantown, WV 26506 USA; 8 Department of Biol- ogy, Colorado State University, Fort Collins, CO 80523 USA; 9 Center for Technology Licensing, Cornell University, Ithaca, NY 14850 USA

**Correction**: ***BMC Plant Biol* 24**,** 766 (2024)**

10.1186/s12870-024-05479-6

Following publication of the original article [1], the author identified minor error in the Funding section. The funding grant of National Institute of General Medical Sciences of the National Institutes of Health is incorrect and needs to be corrected. The change is highlighted in **bold**.

## Current funding

…National Institute of General Medical Sciences of the National Institutes of Health under grant numbers P20GM146584 and P20GM139767.

## Correct funding

…National Institute of General Medical Sciences of the National Institutes of Health under grant numbers P20GM146584 and P20GM13976**9**.

The correction does not compromise the validity of the conclusions and the overall content of the article. The original article [1] has been updated.
