# Supplementary material for: The Defective in Autoregulation (DAR) gene of Medicago truncatula encodes a protein involved in regulating nodulation and arbuscular mycorrhiza
Source: BMC Plant Biol. 2024 Aug 10;24:766. doi: 10.1186/s12870-024-05479-6 (PMC11316349; doi:10.1186/s12870-024-05479-6)
Supplement: Supplementary file 1 — Supplementary Material 1. [file 12870_2024_5479_MOESM1_ESM.pdf]

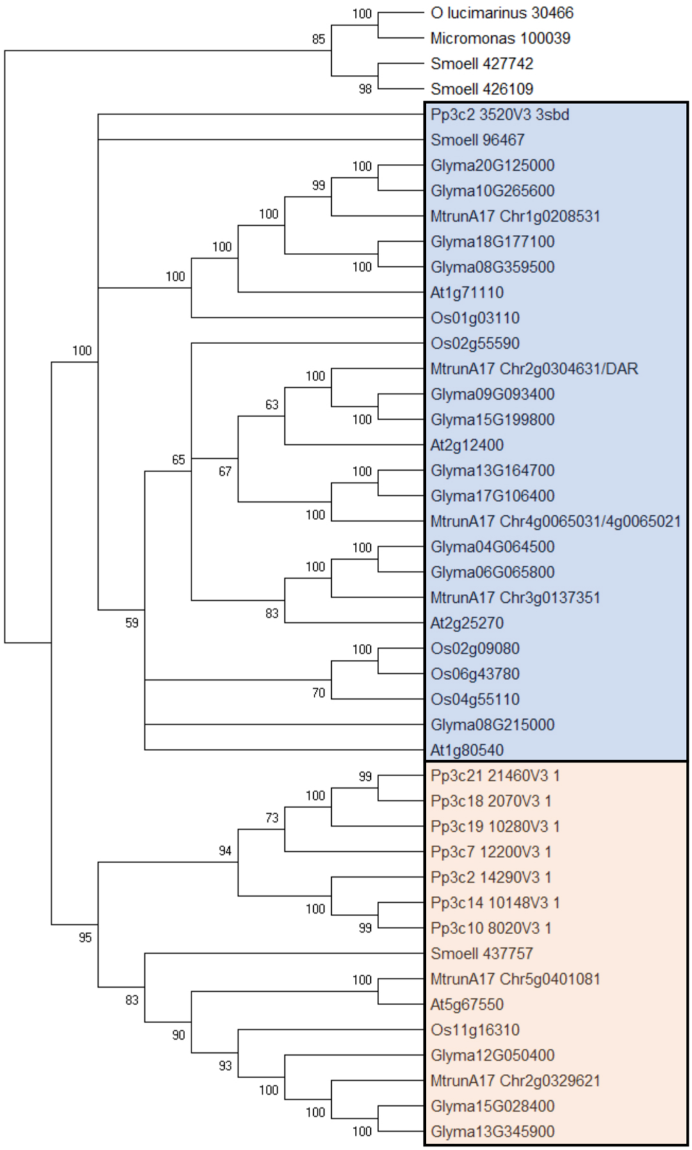


**Additional File 1. Neighbor-joining tree of DAR and related proteins from multiple species.** Included in the tree are proteins from *Medicago truncatula*, Arabidopsis, *Oryza sativa*, *Glycine max*, *Physcomitrella patens*, *Selaginella moellendorffii*, *Micromonas commode* RCC299, and *Ostreococcus lucimarinus*. Gene IDs are shown directly in the consensus tree from 500 bootstrap replicates with the percentage of trees supporting the clustering indicated. Branches supported by less than 50% of the replicates have been collapsed. DAR and the three closest sequences in *M. truncatula* (in Figure 8) are within the cluster shaded in blue. Two additional genes from *M. truncatula* are within the cluster shaded in orange.
