# Supplementary material for: The Defective in Autoregulation (DAR) gene of Medicago truncatula encodes a protein involved in regulating nodulation and arbuscular mycorrhiza
Source: BMC Plant Biol. 2024 Aug 10;24:766. doi: 10.1186/s12870-024-05479-6 (PMC11316349; doi:10.1186/s12870-024-05479-6)
Supplement: Supplementary file 2 — Supplementary Material 2. [file 12870_2024_5479_MOESM2_ESM.pdf]

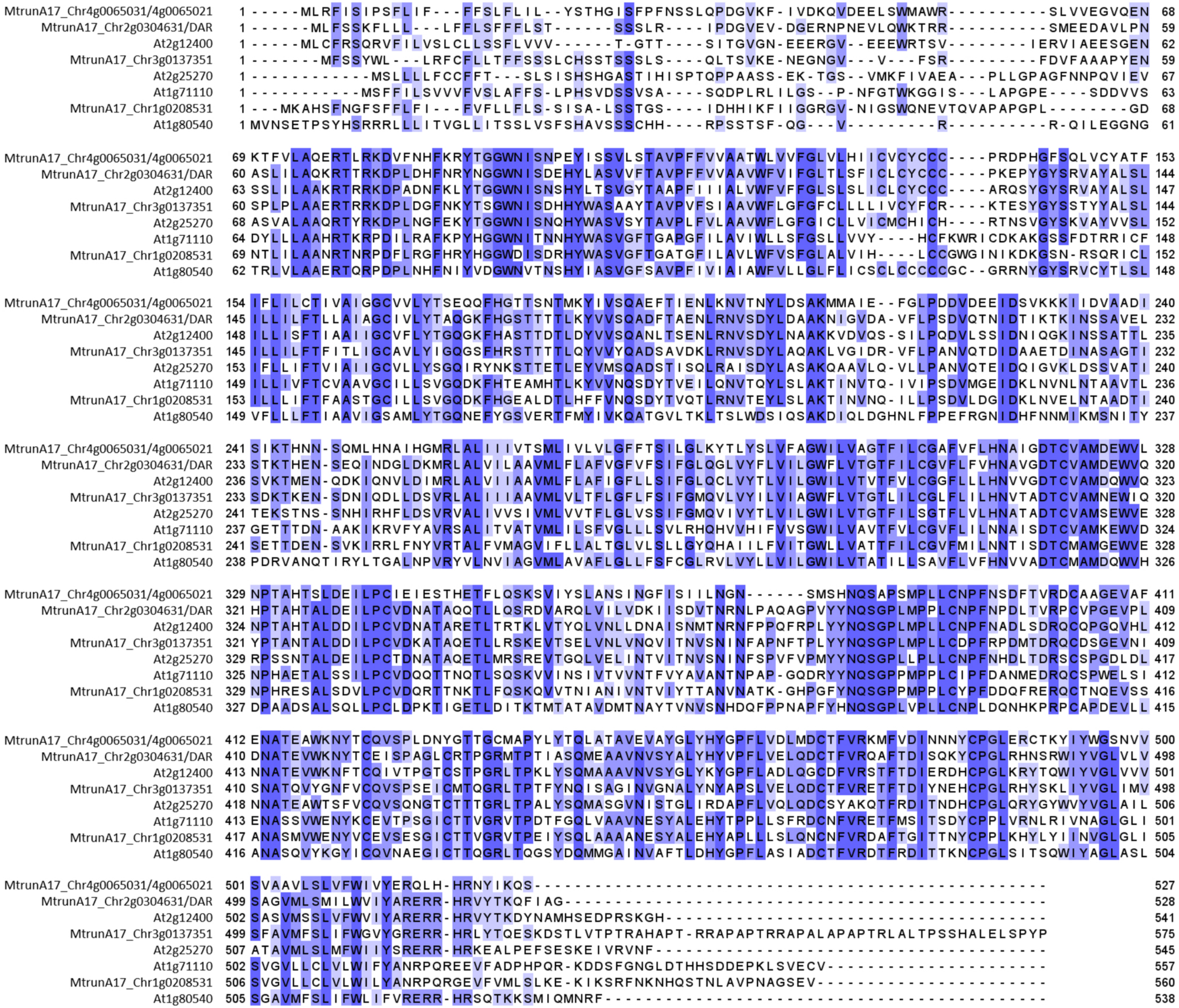

Additional File 2. Clustal Omega alignment of predicted amino acid sequences of DAR and related proteins from *M. truncatula* and *Arabidopsis*. Positions are shaded by weighted percent identity.
