# Supplementary material for: The Defective in Autoregulation (DAR) gene of Medicago truncatula encodes a protein involved in regulating nodulation and arbuscular mycorrhiza
Source: BMC Plant Biol. 2024 Aug 10;24:766. doi: 10.1186/s12870-024-05479-6 (PMC11316349; doi:10.1186/s12870-024-05479-6)
Supplement: Supplementary file 3 — Supplementary Material 3. [file 12870_2024_5479_MOESM3_ESM.pdf]

Additional File 3:primers used in this work

name	sequence 5' to 3'
35S polylinker replacement F	GGTAAAAGCTTTCGACGAATTAATTCCAATC
35S polylinker replacement R	CCTCCGGATCCTTAATTAACTCGAGCGTGTCCTCTCCAAATGAA
DAR-F	CCTCCCTCGAGATGCTTTTCTCCTCTAAATT
DAR-R	CCTCCACTAGTTTAACCAGCAATGAACTGTT
ES50020-A	CACCTTCTTATAACTTCTTCATTG
ES50020-R	GTTTCCAGTTGTCTTCTT
Medtr2g050180-C	TTGGTTCATTTGCCATTCTTCATA
Medtr2g050180-D	AGGCCAACTGATATCCAGCAAGAC
3100	TCACGGGAACATACGAAGAGACG
3114	GCGGACTACAATTTAGAACCATAG
3115	CGTACCTACACATTCATGCGTAAA
3126	ACATGGTTAGTACACATCTCCTAC
3127	ATCGGTTTGTTGTGACACGCTAAT
3130	TCTTTGAATACTCTTTTGCTCGTG
3131	ATTATTGTCAATTGGAACCGTTAT
3132	CATGTGAGATTTCTCCGGCAGGTC
3133	TTTCTGCTGGTGTAATGCTGTCCA
3304	AAACATGCAGAAGTGGGTGGTGAA
3305	AAACGGCGGTAATCGAGAAACAGA
3306	AGGACTCTGTTTGGTTTGGACTAC
3307	TGGAATTCATAAAGACAAAATGAG
3316	TAGCCTAGGCCCATTTCTTATTTA
3317	GAAACATCTGCTCTTACAAGGGC
3327	TATCATGACTGTATTGCACATTAT
3328	GCCCTTGTAAGAGCAGATGTTTC
