# Supplementary material for: The Defective in Autoregulation (DAR) gene of Medicago truncatula encodes a protein involved in regulating nodulation and arbuscular mycorrhiza
Source: BMC Plant Biol. 2024 Aug 10;24:766. doi: 10.1186/s12870-024-05479-6 (PMC11316349; doi:10.1186/s12870-024-05479-6)
Supplement: Supplementary file 4 — Supplementary Material 4. [file 12870_2024_5479_MOESM4_ESM.pdf]

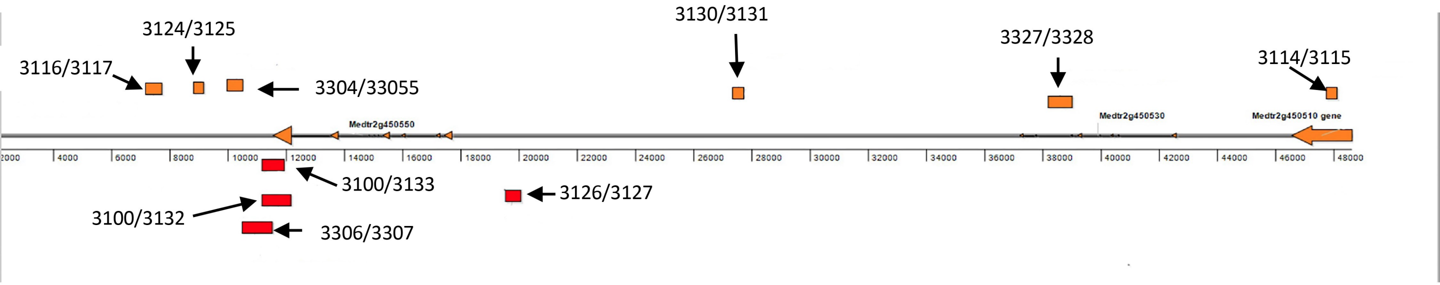

Additional File 4. Diagram of location of primer pairs used to determine deletion in *dar-1* mutant. Positions are indicated in reference in Mt genome 5.0. Primers numbers refer to Additional File 3. Orange PCR products are present in wild type and *dar-1* mutant. Red products were not recovered from *dar-1* mutant but were present in wild type.
